# CTRP6 as a negative regulator of anti-inflammatory M2 macrophage polarization

**DOI:** 10.1097/IN9.0000000000000070

**Published:** 2025-10-27

**Authors:** Jeevotham Senthil Kumar, Emma Kempton, Muhammad Zubair Mehboob, Dingbo Lin, Xia Lei

**Affiliations:** 1 Department of Biochemistry and Molecular Biology, Oklahoma State University, Stillwater, OK, USA; 2 Department of Nutritional Sciences, Oklahoma State University, Stillwater, OK, USA

**Keywords:** CTRP6, adipose tissue, inflammation, negative regulator, M2 macrophage polarization

## Abstract

**Background:**

Chronic low-grade inflammation in adipose tissue, primarily driven by macrophages, plays a central role in obesity pathophysiology. C1q/TNF-related protein 6 (CTRP6), a member of the CTRP family, has emerged as a key regulator of this inflammatory process. Here, we demonstrate that CTRP6 expression is upregulated in adipose tissue macrophages during obesity, where it acts as a potent modulator of macrophage polarization by suppressing M2 polarization.

**Methods:**

In RAW264.7 macrophages, we distinguished M1 and M2 polarization, induced by lipopolysaccharide (LPS) + interferon-gamma (IFNγ) and interleukin (IL)-4, respectively, by selecting two marker genes for each polarization type from a set of five widely used markers, based on a time-course analysis. We then assessed the effects of recombinant CTRP6 protein treatment on M1 and M2 polarization. Finally, we validated our findings in primary bone marrow-derived macrophages (BMDMs).

**Results:**

In naïve RAW264.7 macrophages, recombinant CTRP6 protein upregulated M1 marker genes (*Tnf*, *Nos2*) while downregulating M2 markers (*Mrc1*, *Pparg*). During M1 polarization induced by LPS+IFNγ, CTRP6 treatment had no significant effect. However, during IL-4-induced M2 polarization, CTRP6 not only enhanced M1 markers but also strongly suppressed M2 markers by inhibiting anti-inflammatory signal transducer and activator of transcription 6 (STAT6) signaling and relieving the inhibition of pro-inflammatory ERK1/2 signaling. Additionally, CTRP6 impaired mitochondrial activity, favoring glycolysis in macrophages. Importantly, these effects were serum-independent and confirmed in BMDMs.

**Conclusions:**

Since endogenous CTRP6 expression in BMDMs is upregulated by M1 polarization inducers, it may further hinder inflammation resolution, even in the presence of IL-4 during tissue repair, establishing it as a key driver of adipose tissue inflammation in obesity.

## 1. Introduction

Over the last several decades, the prevalence of obesity has dramatically increased, reaching pandemic dimensions ^[1,2]^. Currently, nearly one-third of the global population is classified as overweight or obese ^[3]^. This chronic condition substantially increases the risk of other noncommunicable diseases, including metabolic disorders, cardiovascular diseases, and certain types of cancer, accounting for over 70% of early deaths worldwide ^[4]^. Furthermore, the economic impact is substantial, with approximately 173 billion dollars expended annually on obesity-related health care, underscoring the limited effectiveness of current treatment modalities ^[5]^.

In the past 20 years, obesity has been increasingly recognized as a trigger of low-grade chronic sterile inflammation in adipose tissue, driven by cytokine release from hypertrophied adipocytes and resident immune cells, primarily macrophages and lymphocytes ^[6–10]^. Macrophages are unique in their capacity to adapt efficiently to changing environments, which is referred to as macrophage polarization, leading them to embrace a spectrum of phenotypes ranging from anti-inflammatory to pro-inflammatory ^[11–13]^. Although no single nomenclature system can effectively define these complex phenotypes, the identification of adipose tissue macrophages as either M1 (classically activated) or M2 (alternatively activated) macrophages has gained prominence. It is widely accepted that in lean adipose tissue, most resident macrophages exhibit an M2 phenotype, playing a crucial role in maintaining adipose tissue homeostasis. In contrast, in obese adipose tissue, there is a significant increase in the number of macrophages, predominantly of the M1 type, contributing to inflammation and insulin resistance ^[14,15]^. M1 macrophages are induced in the presence of inflammatory mediators such as lipopolysaccharide (LPS) and interferon-gamma (IFNγ), leading to the generation of reactive oxygen species (ROS) and the release of inflammatory cytokines such as tumor necrosis factor-α (TNF-α) or interleukin-6 (IL-6). On the other hand, M2 macrophages are generated in response to cytokines such as IL-4 and IL-13, expressing anti-inflammatory factors such as IL-10 and arginase ^[12]^. Given their high plasticity, macrophages dynamically shift between pro- and anti-inflammatory states in response to microenvironmental cues.

Research identifying key regulators of macrophage polarization is essential for understanding the molecular basis of disease progression and developing innovative therapeutic strategies ^[16,17]^. Some medications targeting metabolic diseases associated with obesity have been identified for their role in modulating macrophage polarization. Metformin, a widely used antidiabetic medication, plays a crucial role in modulating macrophage polarization toward the M2 phenotype ^[18,19]^. Additionally, the dipeptidyl peptidase-4 inhibitor Linagliptin, along with the sodium-glucose cotransporter 2 inhibitor Empagliflozin, has shown the ability to reduce obesity-induced inflammation via the phenotypic switch of macrophages ^[20–22]^. Targeting macrophage polarization emerges as a feasible and valuable direction in the development of treatments that could potentially benefit patients with metabolic diseases.

C1q/TNF-related protein 6 (CTRP6), a member of a highly conserved family of 15 secreted proteins ^[23–30]^, plays a significant role in the functional regulation of adipose tissue ^[23,31]^. Studies have demonstrated elevated serum levels of CTRP6 in individuals with obesity and type 2 diabetes compared with healthy individuals ^[32,33]^. Additionally, CTRP6 expression is notably upregulated not only in the adipose tissue of obese human patients but also in diet-induced obese (DIO) mouse models ^[23]^. Mice lacking CTRP6 and fed a high-fat diet (HFD) exhibited reduced inflammation in adipose tissue, evidenced by a substantial decrease in crown-like structure numbers and improved whole-body insulin sensitivity. Intriguingly, both the mRNA expression of the *Tnf-α* gene in adipose tissue and circulating TNF-α protein were significantly decreased in *Ctrp6* KO mice on an HFD. Furthermore, the upregulated expression of CTRP6 was primarily found to originate from the stromal vascular fraction, and CTRP6 can regulate the expression and production of TNF-α in both RAW264.7 macrophage cell line and primary bone marrow-derived macrophages (BMDMs) ^[23]^. A recent study further validated that CTRP6 promotes a pro-inflammatory phenotype, as demonstrated by reduced LPS-induced inflammatory gene expression in CTRP6-deficient BMDMs ^[34]^. However, whether secreted CTRP6 can directly influence the polarization of adjacent naïve macrophages toward a pro-inflammatory M1 or anti-inflammatory M2 phenotype remains an urgent and unresolved question. Moreover, the potential effects of CTRP6 on macrophages during the polarization process, in the presence of M1 or M2 stimuli, have yet to be fully elucidated. In this study, we first identified the primary cell type responsible for the elevated CTRP6 expression in obese individuals using single-cell atlas data analysis. We next investigated the role of CTRP6 in modulating M1 and M2 polarization in the presence or absence of LPS+IFNγ (M1 stimuli) or IL-4 (M2 stimuli) and characterized the associated signaling pathways using RAW264.7 cells. Most importantly, we validated our findings in BMDMs. Our data provide strong evidence supporting the observed reduction in adipose tissue inflammation in *Ctrp6* KO mice.

## 2. Methods

### 2.1 RNA-seq data analyses

For single-cell/nucleus RNA-seq data analysis, we utilized R packages, including Seurat V4 and tidyverse, to visualize the data. The Seurat objects (“.rds” files) for the human white adipose tissue (WAT) atlas were obtained in fully processed form from Emont et al (Single-Cell Portal study no. SCP1376) ^[35]^. The dimensionality, feature, and violin plots presented in our study were created using RStudio (version 2023.06.1+524) running on R (version 4.3.2). The analysis of CTRP6 gene expression changes in the liver and muscle was conducted based on transformed count values from GDS6248 ^[36,37]^ and GDS4015 ^[38]^, respectively. These studies involved microarray analyses performed on livers of C57BL/6J mice fed with HFD for up to 24 weeks and quadriceps muscles of C57BL/6J mice fed with HFD for 56 days.

### 2.2 RAW264.7 macrophage cell culture and treatment

Mouse RAW264.7 macrophages (ATCC TIB-71, a gift from Dr. G. William Wong) were cultured in Dulbecco's modified Eagle's medium (DMEM) (Gibco, Thermo Fisher Scientific, Waltham, MA, USA) supplemented with 10% fetal bovine serum (FBS) (Gibco, Thermo Fisher Scientific, Waltham, MA, USA) and 1% penicillin-streptomycin antibiotics (Gibco, Thermo Fisher Scientific, Waltham, MA, USA). All treatments were performed in 24-well plates with *n* = 5 or 6 for each group, and each of the entire experiment was repeated three times. Before treatment, serum starvation was achieved by a 2-hour incubation in DMEM with 1% FBS. Macrophage polarization was induced by treating cells with 100 ng/mL LPS (00-4976-93, Invitrogen, Thermo Fisher Scientific, Waltham, MA, USA) and 20 ng/mL IFNγ (485-MI-100, R&D Systems, Minneapolis, MN, USA) for M1, and 20 ng/mL IL-4 (404-ML-010, R&D Systems, Minneapolis, MN, USA) for M2. Naïve macrophages (M0) were cultured in medium containing phosphate buffered saline (PBS) with 0.1% BSA (vehicle control), without exposure to M1 or M2 polarization stimuli. For recombinant protein treatment, rCTRP6 protein or vehicle control (Hepes) was added to the culture medium for the indicated treatment period. After treatment, supernatants were collected for enzyme-linked immunosorbent assay (ELISA), and cells were either utilized for RNA extraction or washed with 1× PBS (Corning, Corning, NY, USA) followed by cell lysate preparation.

### 2.3 Recombinant CTRP6 production

Since CTRP6 is a secreted protein, using recombinant protein for in vitro studies offers a clear advantage, as it enables direct assessment of its extracellular effects on target cells under defined conditions, independent of endogenous expression. The recombinant full-length mouse CTRP6 (rCTRP6), incorporating a *C*-terminal FLAG epitope tag (DYKDDDDK), was produced in suspension FreeStyle 293-F cells (Thermo Fisher Scientific, Waltham, MA, USA) at the Mammalian Cell Expression Core of The Johns Hopkins University School of Medicine. The protein was purified and provided by Dr. G. William Wong ^[34]^. Protein concentration was determined using the BCA assay kit (23227, Thermo Fisher Scientific, Waltham, MA, USA), and samples were aliquoted and stored at −80 °C.

### 2.4 RNA extraction and real-time polymerase chain reaction

Total RNA extraction from cells was carried out using TRIzol® reagent (Invitrogen, Thermo Fisher Scientific, Waltham, MA, USA), and reverse transcription was performed using iScript Reverse Transcription (RT) Supermix (Bio-Rad, Hercules, CA, USA) with 500 ng of RNA. Real-time polymerase chain reaction (PCR) analysis was conducted using iTaq Universal SYBR® Green Supermix on a CFX Connect™ system (Bio-Rad, Hercules, CA, USA) with the same amount of RT product from each sample. We tested several internal control genes (*Rplp0*, *Gapdh*, *Ppia*, *β-actin*) and selected the one with the least CT value changes among groups for further analysis. The obtained results were analyzed using the 2^−ΔΔCT^ method ^[39]^. Primer sequences are listed in Table 1.

**Table 1:** Primers for real-time PCR.

Gene	Forward (5′→3′)	Reverse (3′→5′)
*Rplp0*	AGATTCGGGATATGCTGTTGGC	TCGGGTCCTAGACCAGTGTTC
*β-actin*	AGTGTGACGTTGACATCCGTA	GCCAGAGCAGTAATCTCCTTCT
*Ppia*	GAGCTGTTTGCAGACAAAGTTC	CCCTGGCACATGAATCCTGG
*Gapdh*	AGGTCGGTGTGAACGGATTTG	TGTAGACCATGTAGTTGAGGTCA
*Tnf*	CAGGCGGTGCCTATGTCTC	CGATCACCCCGAAGTTCAGTAG
*Il1b*	GCAACTGTTCCTGAACTCAACT	ATCTTTTGGGGTCCGTCAACT
*Il6*	TAGTCCTTCCTACCCCAATTTCC	TTGGTCCTTAGCCACTCCTTC
*Ccl2*	TTAAAAACCTGGATCGGAACCAA	GCATTAGCTTCAGATTTACGGGT
*Nos2*	GTTCTCAGCCCAACAATACAAGA	GTGGACGGGTCGATGTCAC
*Il10*	GCTCTTACTGACTGGCATGAG	CGCAGCTCTAGGAGCATGTG
*Tgfb*	CTCCCGTGGCTTCTAGTGC	GCCTTAGTTTGGACAGGATCTG
*Arg1*	CTCCAAGCCAAAGTCCTTAGAG	AGGAGCTGTCATTAGGGACATC
*Mrc1*	CTCTGTTCAGCTATTGGACGC	CGGAATTTCTGGGATTCAGCTTC
*Pparg*	GGAAGACCACTCGCATTCCTT	GTAATCAGCAACCATTGGGTCA

### 2.5 ELISA and Western blot analysis

Mouse TNF-α ELISA kit (MTA00B, R&D Systems, Minneapolis, MN, USA) was used to quantify the secreted protein in the cell culture supernatant following the manufacturer’s instruction. For Western blot, whole cell lysates were prepared by lysing PBS-washed cells in the radioimmunoprecipitation assay (RIPA) Lysis and Extraction Buffer (PI89901, Thermo Fisher Scientific, Waltham, MA, USA) supplemented with protease inhibitors (A32953, Thermo Fisher Scientific, Waltham, MA, USA) and phosphatase inhibitors (PhosSTOP™, Roche, Basel, Switzerland). The total protein concentration was determined using the BCA protein assay kit (23227, Thermo Fisher Scientific, Waltham, MA, USA). The lysates were then processed in 4× Laemmli sample buffer (1610747, Bio-Rad, Hercules, CA, USA) and boiled at 95 °C for 5 minutes for electrophoresis. Equal amounts of total protein lysates were loaded on Mini-PROTEAN Precast Protein Gels (Bio-Rad, Hercules, CA, USA), and subsequently, the proteins were transferred to a nitrocellulose membrane using the Trans-Blot® Turbo™ transfer system (Bio-Rad, Hercules, CA, USA). Protein bands were developed using the ECL substrate (1705061, Bio-Rad, Hercules, CA, USA) and visualized by ChemiDoc™ (Bio-Rad, Hercules, CA, USA) followed by densitometric quantification using the ImageJ software (National Institutes of Health [Bethesda, MA, USA] and the Laboratory for Optical and Computational Instrumentation, University of Wisconsin [Madison, WI, USA]). The antibodies used for blotting are listed in Table 2.

**Table 2.** Antibodies used in Western blot.

Antibody	Catalog no.	Manufacturer	Dilution
MRC1	ab64693	Abcam plc.	1:500
β-actin	4970S	Cell Signaling Technology®	1:5000
phospho-AKT	9271S	Cell Signaling Technology®	1:500
AKT	9272S	Cell Signaling Technology®	1:1000
phospho-STAT6	56554S	Cell Signaling Technology®	1:500
STAT6	5397S	Cell Signaling Technology®	1:1000
phospho-ERK1/2 (p44/42)	9101S	Cell Signaling Technology®	1:1000
ERK1/2 (p44/42)	9102S	Cell Signaling Technology®	1:1000
phospho-p38 MAPK	9211S	Cell Signaling Technology®	1:1000
p38 MAPK	9212S	Cell Signaling Technology®	1:1000
Goat anti-rabbit IgG secondary antibody, HRP	31460	Thermo Scientific Inc.	1:10,000

AKT, protein kinase B; HRP, horseradish peroxidase; MAPK, mitogen-activated protein kinase; MRC1, mannose receptor C-type 1; STAT6, signal transducer and activator of transcription 6.

### 2.6 ATP (adenosine triphosphate) production rate analysis

The real-time ATP production rate assay was performed on the Seahorse XFe96 system (Agilent Technologies, Santa Clara, CA, USA) using its kit (103592-100). Briefly, RAW264.7 macrophages were seeded in V3 PS cell culture microplates (101085-004) at 2.5 × 10^4^ cells/well. After 24 hours, cells were treated with different doses of rCTRP6 (0, 250, 500, and 1000 ng/mL) for an additional 24 hours (*n* = 15/group). At the end of the treatment period, the oxygen consumption rate and extracellular acidification rate were measured under basal conditions, followed by the sequential addition of 1.5 μM oligomycin A (75351-5MG, Sigma-Aldrich, St. Louis, MO, USA) and 0.5 μM rotenone (557368-1GM, Sigma-Aldrich, St. Louis, MO, USA) + 0.5 μM antimycin A (A8674-25MG, Sigma-Aldrich, St. Louis, MO, USA) to selectively inhibit mitochondrial respiration and glycolysis respectively. The results were analyzed with the Wave (v2.6.3) software (Agilent Technologies, Santa Clara, CA, USA), and ATP production rates were calculated using the Seahorse XF Real-time ATP rate assay report generator.

### 2.7 Fluorescent microscopy

The RAW264.7 macrophages (5 × 10^4^/well) were seeded onto Lab-Tek™ II Chamber Slide (154534, Thermo Fisher Scientific, Waltham, MA, USA). The following day, cells were treated with or without IL-4 (20 ng/mL) along with or without rCTRP6 (1000 ng/mL) for 24 hours. Mitochondrial activity in the macrophages was visualized by incubating the cells in 200 nM MitoTracker® Red CM-H_2_Xros stain (M7513, Thermo Fisher Scientific, Waltham, MA, USA) for 30 minutes. A 5-μM solution of Hoechst 33342 (PI62249, Thermo Fisher Scientific, Waltham, MA, USA) was used to stain DNA as a nuclear counterstain. The images were captured using the EVOS M5000 (Thermo Fisher Scientific, Waltham, MA, USA) fluorescent microscope.

### 2.8 Primary BMDMs culture and treatment

Bone marrow cells were isolated from the femurs and tibiae of C57BL/6 mice and cultured on Petri dishes in RPMI 1640 medium (Gibco, Thermo Fisher Scientific, Waltham, MA, USA) supplemented with 20% FBS (Gibco, Thermo Fisher Scientific, Waltham, MA, USA), 20 ng/mL macrophage colony-stimulating factor (416-ML-010, R&D Systems, Minneapolis, MN, USA), 1% penicillin-streptomycin (Gibco, Thermo Fisher Scientific, Waltham, MA, USA), and 2 mM GlutaMAX (Gibco, Thermo Fisher Scientific, Waltham, MA, USA). Differentiated cells (BMDMs) were harvested on day 8 for further experiments. BMDMs were seeded and incubated for 4 hours at 37 °C with 5% CO_2_, followed by polarization either with 100 ng/mL LPS and 20 ng/mL IFNγ for M1 polarization or 20 ng/mL IL-4 for M2 polarization over 24 hours. Concurrently, varying doses of rCTRP6 protein were added to the culture medium. After treatment, supernatants were collected for ELISA or Griess assay, and cells were either processed for RNA extraction or washed with 1× PBS (Corning, Corning, NY, USA) followed by cell lysate preparation.

### 2.9 Nitric oxide production assay

The nitric oxide (NO) production in BMDMs was assessed by quantifying nitrite concentrations in the culture supernatant using Griess reagent kit (Invitrogen, Thermo Fisher Scientific, Waltham, MA, USA). Briefly, 50 μL of BMDM supernatants were incubated with Griess reagent (0.1% *N*-(1-naphthyl) ethylenediamine dihydrochloride and 1% sulfanilic acid in 5% phosphoric acid) for 30 minutes at room temperature. Absorbance was then measured at 548 nm, and nitrite concentrations were extrapolated from a standard curve generated using nitrite standard solutions.

### 2.10 Statistical analysis

A two-tailed Student’s *t*-test was used for comparison between two groups, and a one-way analysis of variance (ANOVA) was used for multi-group comparison, followed by either Dunnett’s post hoc test (rCTRP6 dose of 0 as control group) or Tukey’s post hoc test (all groups compared with each other). Two-way ANOVA followed by Bonferroni’s multiple comparison test was used in the time-course comparison. Time-course data and Seahorse data are reported as mean ± standard deviation (SD). All other data are presented as mean ± standard error of the mean (SEM). *P* <0.05 was considered statistically significant.

### 2.11 Ethics statement

This study reanalyzed previously published human data obtained from Single-Cell Portal (study no. SCP1376) with permission and no additional Institutional Review Board (IRB) approval was required. In the original study, human subcutaneous and visceral adipose tissue samples were collected under protocols approved by Open IRBs (the Beth Israel Deaconess Medical Center Committee on Clinical Investigations (IRB #2011P000079), and the University of Pittsburgh Medical Center (STUDY #19010309)). All animal experiments were approved by the Institutional Animal Care and Use Committee of Oklahoma State University (IACUC-20-33) and conducted in accordance with the National Institute of Health guidelines and followed the standards established by the Animal Welfare Acts.

## 3. Results

### 3.1 Macrophages are the predominant source of CTRP6 in obese adipose tissue

**Figure 1.**
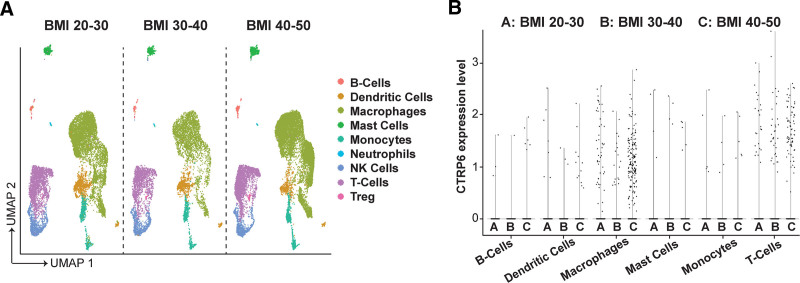
**Single-cell atlas of human white adipose tissue (WAT) reveals increased CTRP6 expression in macrophages during obesity.** (A) Uniform manifold approximation and projection (UMAP) of all immune cells in WAT stratified by body mass index (BMI). Data were retrieved from the human WAT atlas, generated from 166,149 total cells (28,465 single cells and 137,684 single nuclei) derived from subcutaneous and visceral adipose tissue of individuals with different BMI (Single Cell Portal study no. SCP1376). (B) Violin plot illustrates CTRP6 expression in various types of immune cells in WAT, categorized by BMI.

In the previous study, we observed a significant alteration in CTRP6 expression within the adipose tissue of obese mice, pinpointing its association with the stromal vascular fraction. Here, leveraging the recent detailed human WAT atlas by Emont et al at a single-cell resolution across varying body mass index (BMI) ^[35]^, we aimed to elucidate the source of elevated CTRP6 expression. Analyzing integrated data from 28,465 single cells and 137,684 single nuclei extracted from human WAT across a BMI spectrum of 20 to 50, we initially identified distinct immune cell types (Figure 1A). Notably, our analysis highlighted macrophages as the primary contributors to the augmented CTRP6 expression, followed by T-cells (Figure 1B). Given the pivotal role of macrophage-mediated inflammation in the pathology of obesity, it becomes imperative to explore whether CTRP6 exerts any effect on macrophage function, particularly M1/M2 polarization.

### 3.2 Gene expression profiles during macrophage polarization are characterized

**Figure 2.**
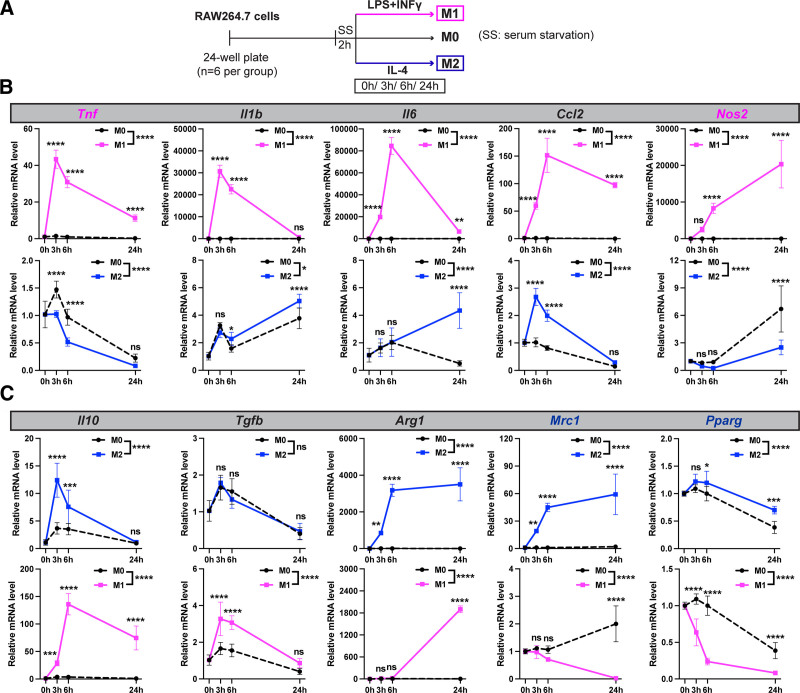
**Time-course real-time PCR analysis of marker genes associated with M1/M2 macrophage polarization in RAW264.7 cells.** (A) Experimental timeline for the induction of RAW264.7 macrophage polarization. Cells were seeded into 24-well plates and, after 24 hours, were serum-starved by incubation in DMEM with 1% FBS for 2 hours. Macrophage polarization was then induced by treating the cells for 24 hours with 100 ng/mL LPS+20 ng/mL IFNγ for M1 polarization, or 20 ng/mL IL-4 for M2 polarization, or vehicle control for naïve state M0. Cell samples were collected before treatment (0 hours) and at 3, 6, and 24 hours post-treatment. RNA was extracted, followed by reverse transcription and real-time PCR analysis. (B) Expression comparison of widely used M1 marker genes (*Tnf*, *Il1b*, *Il6*, *Ccl2*, *Nos2*) during M1 polarization (upper panel) vs M2 polarization (lower panel) (*n* = 6/group at each time point). (C) Expression comparison of widely used M2 marker genes (*Il10*, *Tgfb*, *Arg1*, *Mrc1*, *Pparg*) during M2 polarization (upper panel) vs M1 polarization (lower panel) (*n* = 6/group at each time point). Expression levels are normalized to *Rplp0* gene in each sample. Data are shown as the mean ± SD. Two-way ANOVA followed by Bonferroni’s multiple comparison test. *P < 0.05; **P < 0.01; ***P < 0.001; ****P < 0.0001. ANOVA, analysis of variance; DMEM, Dulbecco's modified Eagle's medium; FBS, fetal bovine serum; IFNγ, interferon-gamma; LPS, lipopolysaccharide; PCR, polymerase chain reaction; SD, standard deviation.

To assess the impact of CTRP6 on macrophage polarization, we first established an in vitro cell model using the RAW264.7 macrophage cell line and performed real-time PCR to scrutinize the expression of well-established marker genes at different times of polarization induced by LPS+IFNγ for M1 polarization and IL-4 for M2 polarization (Figure 2A). Primer sequences are listed in Table 1. For M1 polarization, the marker genes *Tnf*, *Il1b*, *Il6*, *Ccl2*, and *Nos2* were examined (Figure 2B). Inducing M1 polarization predictably resulted in a swift and substantial increase in all markers. The expression of genes encoding the inflammatory cytokines *Tnf*, *Il1b*, and *Il6* significantly decreased after the initial hours of induction, suggesting a potent and acute mode of action. On the other hand, *Ccl2* and *Nos2*, indicative of macrophage migratory ability and arginine metabolism, respectively, exhibited sustained high expression levels, highlighting their prolonged necessity in the M1 phenotype. As anticipated, inducing M2 polarization did not have as significant an effect on any of the M1 marker genes. However, IL-4 mildly but significantly induced the expression of *Il1b*, *Il6*, and *Ccl2*, which were excluded from the M1 marker genes in our study. Overall, the expression profiles of *Tnf* and *Nos2*, especially at the 24 hours time point, collectively depicted a discernible trend distinguishing the M1 and M2 phenotypes of the macrophages.

While *Il10*, *Tgfb*, *Arg1*, *Mrc1*, and *Pparg* have been commonly employed as M2 markers in previous studies, M2 induction with IL-4 yielded distinct effects on these five genes in our model (Figure 2C). *Il10* and *Tgfb* exhibited less significant or no induction, respectively, contrasting with their unexpected significant and minor induction during M1 polarization. *Arg1* displayed a significant induction during both M1 and M2 polarization, rendering it less suitable as a distinctive marker for these processes. In contrast, the expressions of *Mrc1*, the gene encoding a mannose receptor protein (mannose receptor C-type 1, MRC1) responsible for clearing pathological glycoproteins, and *Pparg*, a pivotal adipogenic and anti-inflammatory transcription factor, distinctly delineate the differences between the two macrophage phenotypes. Collectively, these four genes (*Tnf*, *Nos2*, *Mrc1*, and *Pparg*) serve as a benchmark for assessing the impact of CTRP6 on macrophage polarization phenotypes.

### 3.3 Modulation of recombinant CTRP6 protein on macrophage polarization

**Figure 3.**
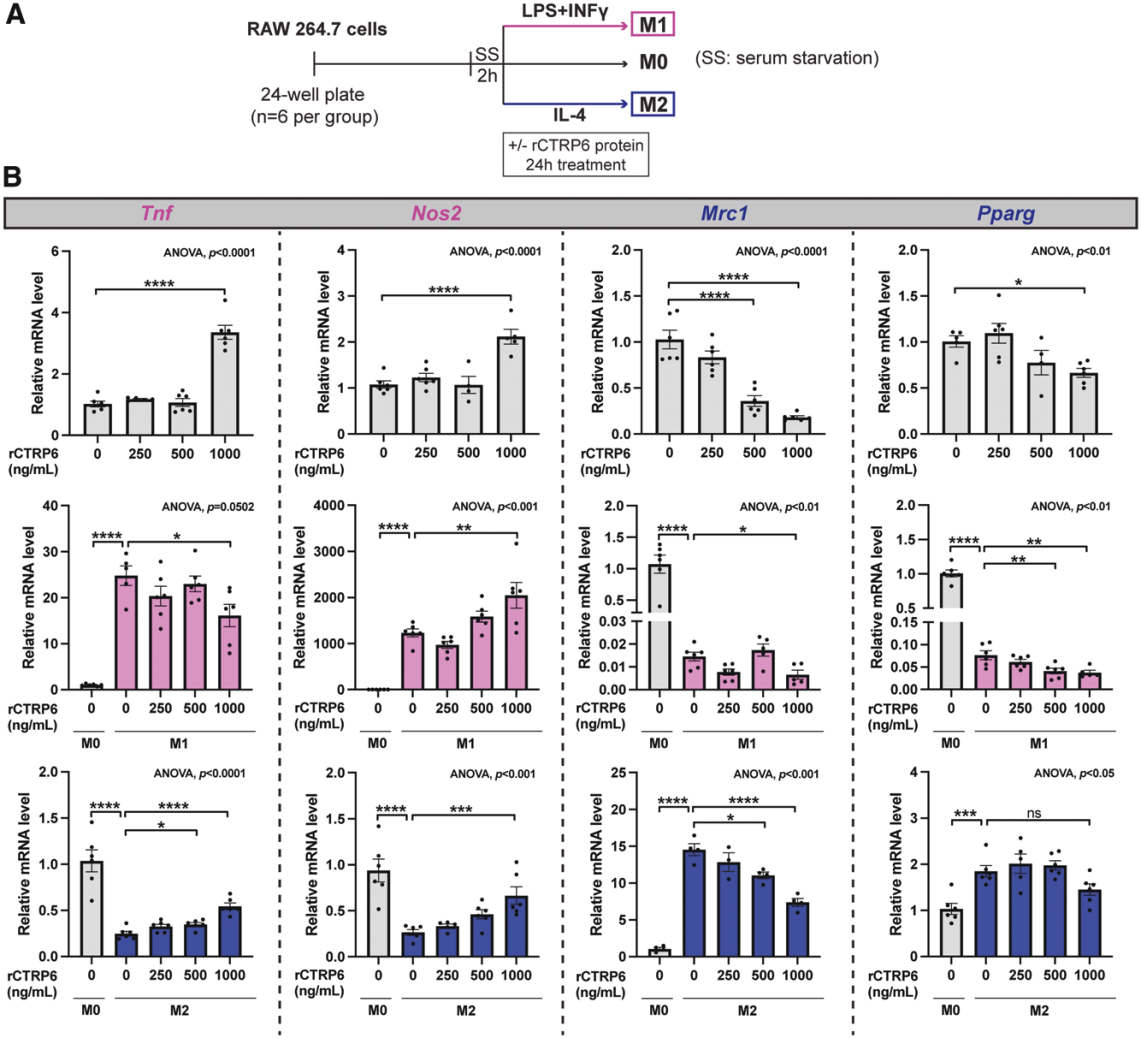
**Recombinant CTRP6 protein (rCTRP6) treatment in RAW264.7 macrophages with or without polarization inducers.** (A) Experimental timeline for rCTRP6 protein treatment in conjunction with macrophage polarization. Cells were seeded into 24-well plates and, after 24 hours, were serum-starved by incubation in DMEM with 1% FBS for 2 hours. Macrophage polarization was then induced by treating the cells for 24 hours with 100 ng/mL LPS+20 ng/mL IFNγ for M1 polarization, or 20 ng/mL IL-4 for M2 polarization, or vehicle control for naïve state M0. Different concentrations of rCTRP6 (0, 250, 500, and 1000 ng/mL) were applied under each condition. (B) Expression of selected M1 and M2 marker genes in response to the indicated doses of rCTRP6 protein (*n* = 6/group) in M0, M1, and M2 states. Expression levels are normalized to *Gapdh*, *Ppia*, and *Rplp0* in M0, M1, and M2 states, respectively. Data are shown as the mean ± SEM. Two-tailed Student’s *t*-tests for two-group comparison (M0 vs M1 without rCTRP6; M0 vs M2 without rCTRP6) and One-way ANOVA for multi-group comparison (*P* value indicated in the upper-right corner of the figure) followed by Dunnett’s post hoc test to determine rCTRP6’s effect. *P < 0.05; **P < 0.01; ***P < 0.001; ****P < 0.0001. ANOVA, analysis of variance; DMEM, Dulbecco's modified Eagle's medium; FBS, fetal bovine serum; LPS, lipopolysaccharide; SEM, standard error of the mean.

Naïve macrophages were exposed to increasing doses of purified mouse recombinant CTRP6 protein (rCTRP6) along with M1 and M2 polarization inducers, respectively (Figure 3A). In naïve macrophages (M0) (Figure 3B, upper panel), rCTRP6 significantly induced *Tnf* and *Nos2* expression while dose-dependently suppressing *Mrc1* and mildly reducing *Pparg* expression, indicating a robust dual effect of CTRP6 on macrophage polarization. During M1 polarization (Figure 3B, middle panel), rCTRP6 treatment further enhanced *Nos2* expression, without a significant effect on *Tnf* expression. Both of these M1 marker genes have been dramatically induced by LPS+IFNγ. Interestingly, rCTRP6 slightly intensified the suppression of *Mrc1* and *Pparg* expression during M1 induction. On the other hand, during M2 polarization (Figure 3B, lower panel), IL-4 markedly downregulated the M1 markers *Tnf* and *Nos2*, and rCTRP6 dose-dependently restored their expression. Importantly, rCTRP6 prominently dose-dependently suppressed the upregulation of *Mrc1*, with only marginal effects observed on *Pparg*. Collectively, our data indicate that CTRP6 has the potential to promote pro-inflammatory M1 polarization and exerts a more pronounced impact on the suppression of anti-inflammatory M2 polarization.

**Figure 4.**
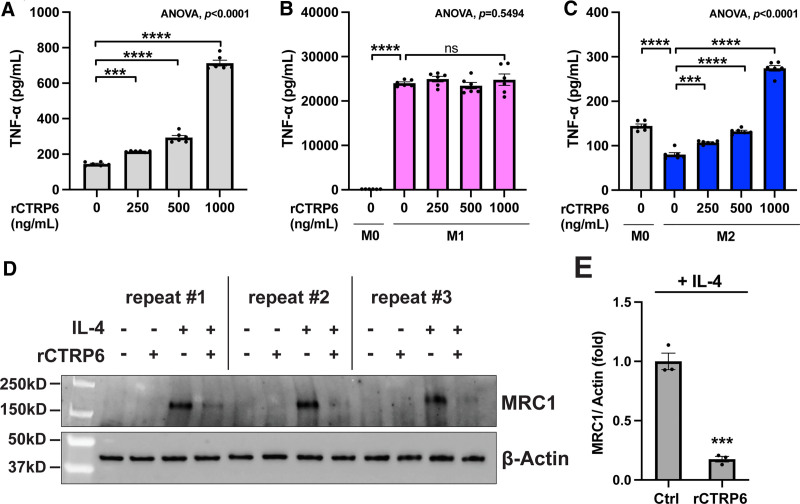
**rCTRP6 protein treatment modulates the expression of marker genes involved in the polarization of RAW264.7 macrophages at the protein level.** (A–C) TNF-α production in macrophages treated with indicated doses of rCTRP6 protein (*n* = 6/group) was examined by ELISA in M0, M1, and M2 states, respectively. Data are shown as the mean ± SEM. Two-tailed Student’s *t*-tests for two-group comparison (M0 vs M1 without rCTRP6; M0 vs M2 without rCTRP6) and One-way ANOVA for multi-group comparison (*P* value indicated in the upper-right corner of the figure) followed by Dunnett’s post hoc test to determine rCTRP6’s effect. ***P < 0.001; ****P < 0.0001. (D) Western blot analysis of MRC1 protein in whole cell lysates from naïve M0 state (without IL-4) and polarized M2 state (with IL-4) in conjunction with a 24-hour treatment of 1000 ng/mL rCTRP6. MRC1 was not detected in the M0 state. The experiment was repeated three times. (E) Densitometric analysis for Western blot results from M2 state using ImageJ software (*n* = 3/group). β-actin is used as the internal control. Data are shown as the mean ± SEM. Two-tailed Student’s *t*-tests for two-group comparison. ***P < 0.001. ANOVA, analysis of variance; ELISA, enzyme-linked immunosorbent assay; IL-4, interleukin-4; MRC1, mannose receptor C-type 1; SEM, standard error of the mean; TNF-α, tumor necrosis factor-α.

We further evaluated CTRP6-modulated macrophage phenotypes at the protein level. By quantifying the concentration of secreted TNF-α in the cell culture supernatants of macrophages, we observed a significant upregulation of TNF-α levels with increasing doses of rCTRP6 in both naïve macrophages (Figure 4A) and during M2 polarization (Figure 4C). No additional impact of rCTRP6 on TNF-α production was noted during M1 polarization, following the robust effects of LPS+IFNγ (Figure 4B), consistent with our results from real-time PCR. Furthermore, probing whole cell lysates of naïve and M2 macrophages for the MRC1 protein revealed that rCTRP6 significantly reduced the levels of this protein during M2 polarization, even though it is dramatically induced by IL-4 (Figure 4D,E). In summary, our results indicate that CTRP6 is involved in the observed dampened anti-inflammatory phenotype in M2 macrophages within obese adipose tissue, which is a critical aspect for resolving adipose tissue inflammation.

### 3.4 Distinct signaling pathways are involved in CTRP6-modulated macrophage polarization

**Figure 5.**
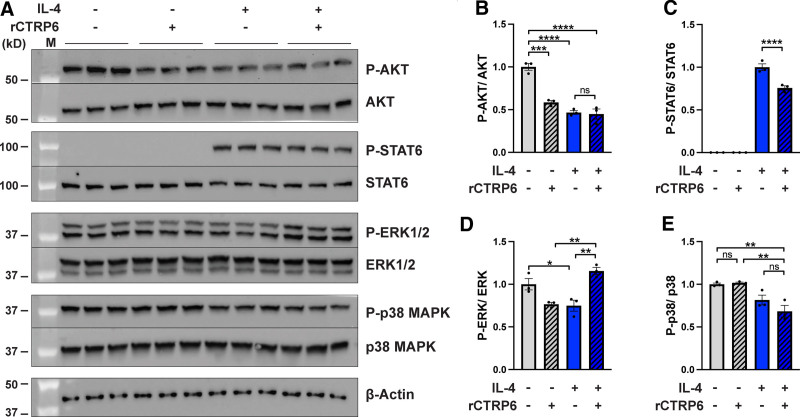
**Distinct signaling pathways are involved in CTRP6-modulated macrophage polarization.** (A) Western blot analysis of the phosphorylation status of AKT, STAT6, ERK1/2, and p38 MAPK proteins in naïve M0 and polarized M2 macrophages, with and without treatment with rCTRP6 protein (1000 ng/mL) for 24 hours. Each condition had three replicates. (B–E) Densitometric analysis of Western blot results for phosphorylation status relative to total protein, conducted using ImageJ software. Data are shown as the mean ± SEM. One-way ANOVA followed by Tukey’s post hoc test. *P < 0.05; **P < 0.01; ***P < 0.001, ****P < 0.0001. ANOVA, analysis of variance; SEM, standard error of the mean.

To identify the signaling pathways contributing to the impact of CTRP6 on macrophage polarization, we examined the phosphorylation status of proteins involved in major signaling pathways during macrophage polarization with or without rCTRP6 treatment. During short-term treatment (30 and 60 minutes), rCTRP6 did not significantly alter the signaling pathways tested (data not shown). However, notable changes were observed with extended treatment (24 hours) (Figure 5A). The phosphorylation of signal transducer and activator of transcription 6 (STAT6) was markedly induced by IL-4 treatment, in stark contrast to the undetectable levels observed in naïve macrophages. However, rCTRP6 significantly reduced this anti-inflammatory signaling (Figure 5C). Simultaneously, rCTRP6 enhanced the phosphorylation of p44/42 MAPK (ERK1/2), which was suppressed by IL-4 treatment (Figure 5D). Collectively, the suppression of STAT6 signaling and the restoration of ERK1/2 signaling could contribute to the dampened M2 polarization by CTRP6 in the context of IL-4. Notably, in naïve macrophages, rCTRP6 dramatically inhibited the AKT pathway (Figure 5B). A similar extent of inhibition was also observed with IL-4 treatment, suggesting that the AKT pathway may not be responsible for the dampened M2 polarization, but could be involved in promoting M1 polarization observed in the naïve state without IL-4.

### 3.5 Recombinant CTRP6 protein alters macrophage energy metabolism

**Figure 6.**
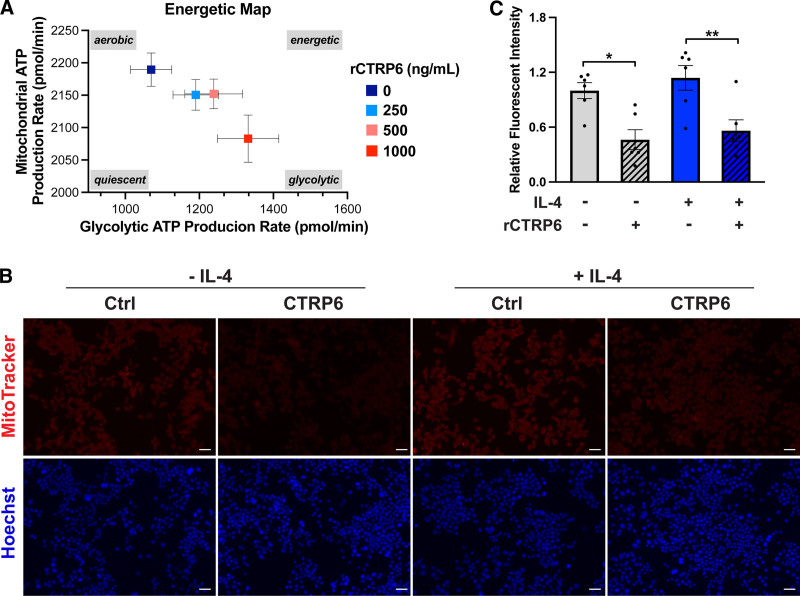
**rCTRP6 protein treatment promotes glycolysis and reduces mitochondrial activity in RAW264.7 macrophages.** (A) The energetic map from Seahorse XF extracellular flux analysis shows ATP production rates in macrophages treated with the indicated doses of rCTRP6 protein for 24 hours, highlighting their respiratory status (*n* = 15/group). Data are shown as the mean ± SD. (B) Representative fluorescent microscopy images of macrophages in both naïve and M2 polarization states, with and without rCTRP6 protein treatment (1000 ng/mL, 24 hours). Mitochondria activities are visualized using MitoTracker® Red CM-H_2_Xros, and DNA is stained with Hoechst 33342 dye. Scale Bar = 30 μm. (C) Fluorescent intensity was measured by calculating the corrected total cell fluorescence (CTCF) using ImageJ software. Relative fluorescent intensity was determined by calculating the ratio to the naïve state without rCTRP6 treatment. Data are shown as the mean ± SEM. One-way ANOVA followed by Tukey’s post hoc test. *P < 0.05; **P < 0.01. ANOVA, analysis of variance; ATP, adenosine triphosphate.

Although not mutually exclusive, M1 macrophages predominantly depend on glycolysis, while M2 macrophages primarily utilize mitochondrial oxidative phosphorylation (OXPHOS) to meet their energetic requirements ^[40]^. To explore the impact of rCTRP6 on macrophage metabolism, we conducted a real-time ATP rate assay using the Seahorse XF system on naïve macrophages treated with increasing concentrations of rCTRP6. The results revealed a shift in macrophage energy metabolism, as shown in the energetic map, with an increased reliance on glycolysis to meet ATP demands at higher concentrations of rCTRP6 (Figure 6A). Concurrently, ATP supply from mitochondrial OXPHOS trended downwards, suggesting that rCTRP6 restricts mitochondrial activity.

To further validate this observation, we used the reduced MitoTracker® Red CM-H_2_Xros dye to stain active mitochondria in both naïve and M2 macrophages, with and without rCTRP6 treatment (Figure 6B). The results showed that rCTRP6 treatment significantly decreased mitochondrial activity in both naïve macrophages and polarized M2 macrophages (Figure 6C), providing additional support for the robust inhibitory effect of CTRP6 on M2 polarization and its impact on the anti-inflammatory phenotype of macrophages.

### 3.6 Effect of CTRP6 is validated under different culture conditions

**Figure 7.**
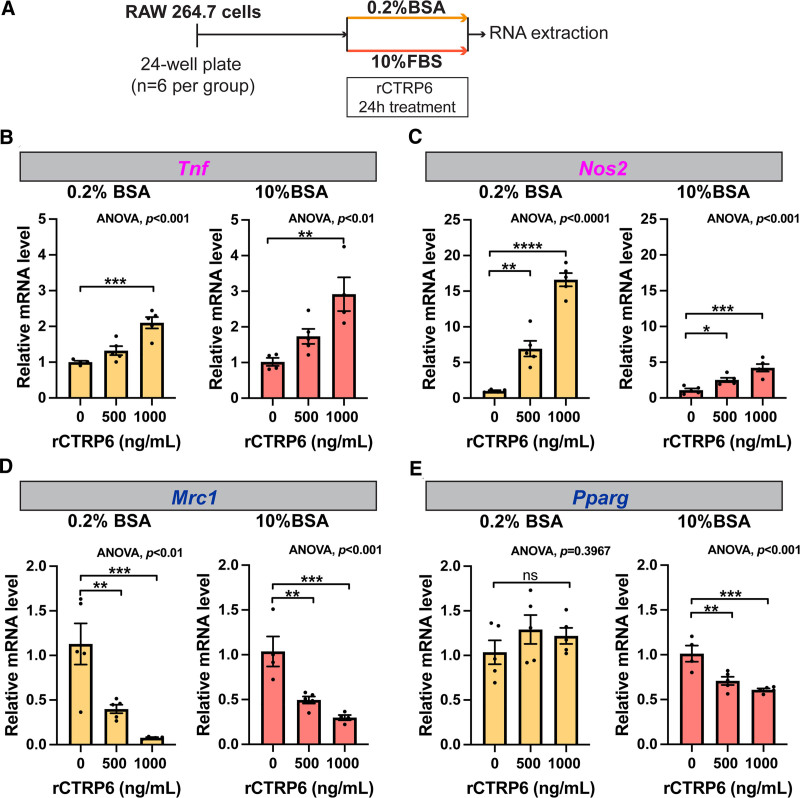
**Effects of rCTRP6 protein on RAW264.7 macrophages are not serum dependent.** (A) Experimental timeline for rCTRP6 treatment with either 0.2% BSA or 10% FBS in the culture medium. Cells were seeded into 24-well plates and, after 24 hours, treated with rCTRP6 in either 0.2% BSA or 10% FBS medium for 24 hours. Different concentrations of rCTRP6 (0, 500, 1000 ng/mL) were applied under each condition. (B) Expression levels of M1 polarization marker genes (*Tnf*, *Nos2*) and M2 polarization marker genes (*Mrc1*, *Pparg*) in response to the indicated doses of rCTRP6 protein under the two different culture conditions (*n* = 5/group). Expression levels are all normalized to *Rplp0* in each group. Data are shown as the mean ± SEM. One-way ANOVA followed by Dunnett’s post hoc test to determine rCTRP6’s effect. *P < 0.05; **P < 0.01; ***P < 0.001; ****P < 0.0001. ANOVA, analysis of variance; FBS, fetal bovine serum; SEM, standard error of the mean.

As cell phenotypes can be influenced by diverse culture conditions, we examined the potential impact of serum concentration on our results. In our model system, we opted for the inclusion of 10% FBS to meet the high serum concentration requirements for cytokine release ^[41]^. To thoroughly assess the influence of serum concentration, we conducted a direct comparison between a serum-free system (0.2% BSA) and our standard system (Figure 7A). In the absence of serum in the culture media, the expression profiles of *Tnf* and *Mrc1* remained unaffected, indicating that the absence of serum did not alter the regulation of these genes by CTRP6 (Figure 7B,D). In the case of *Nos2*, while the overall trends remained consistent, the expression levels were amplified in the absence of serum (Figure 7C). Notably, the impact on *Pparg* expression was not observed in the serum-free system (Figure 7E). These findings collectively reinforce the role of CTRP6 in modulating macrophage M1/M2 polarization. The lack of significant variations in the absence of serum further strengthens the robustness and consistency of these effects.

### 3.7 Effect of CTRP6 is validated in the primary BMDMs

**Figure 8.**
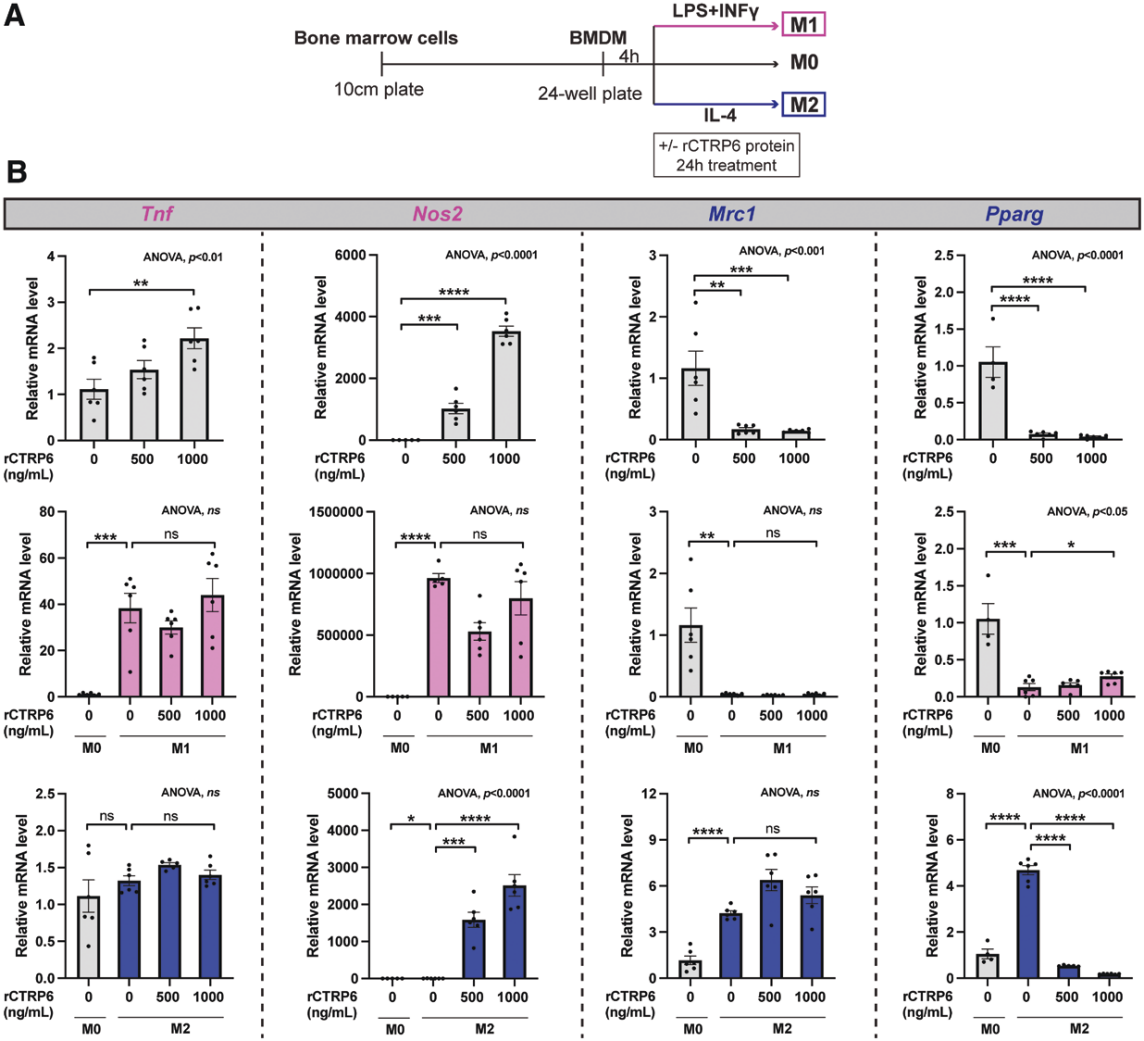
**Effects of rCTRP6 protein in primary BMDMs with or without polarization inducers.** (A) Experimental timeline for rCTRP6 protein treatment in conjunction with macrophage polarization. Bone marrow cells were plated into 10-cm plates, differentiated for 8 days, and then BMDMs were collected and plated into 24-well plates. Four hours later, they were treated with different concentrations of rCTRP6 along with M1 inducers (100 ng/mL LPS+20 ng/mL IFNγ) or M2 inducers (20 ng/mL IL-4) or without inducers (M0). (B) Expression of selected M1 and M2 marker genes in response to the indicated doses of rCTRP6 protein (*n* = 6/group) in naïve M0, M1, and M2 states. Expression levels are normalized to *Ppia*. Data are shown as the mean ± SEM. Two-tailed Student’s *t*-tests for two-group comparison (M0 vs M1 without rCTRP6; M0 vs M2 without rCTRP6) and One-way ANOVA for multi-group comparison (*P* value indicated in the upper-right corner of the figure) followed by Dunnett’s post hoc test to determine rCTRP6’s effect. *P < 0.05; **P < 0.01; ***P < 0.001; ****P < 0.0001. ANOVA, analysis of variance; BMDMs, bone marrow-derived macrophages; IFNγ, interferon-gamma; IL-4, interleukin-4; LPS, lipopolysaccharide.

While RAW264.7 cells are a widely used immortalized cell line for *in vitro* studies, primary BMDMs more accurately represent in vivo macrophage behavior. Therefore, we examined the effects of CTRP6 in primary BMDMs. After deriving BMDMs from bone marrow cells, they were treated with or without rCTRP6 protein in non-polarized (M0) or polarized (M1 or M2) states, similar to the treatment in RAW264.7 cells (Figure 8A). In the M0 state, *Tnf* expression was induced by rCTRP6 to a similar extent as observed in RAW264.7 cells. However, *Nos2* was significantly upregulated, and *Mrc1* and *Pparg* were downregulated to a much greater degree in BMDMs (Figure 8B, upper panel). In the M1 state, these three genes showed more pronounced responses to M1 polarization, with no significant changes upon rCTRP6 protein treatment (Figure 8B, middle panel). In the M2 state, rCTRP6 markedly enhanced *Nos2* expression and suppressed *Pparg* expression, with minimal effects on *Tnf* and *Mrc1* (Figure 8B, lower panel).

**Figure 9.**
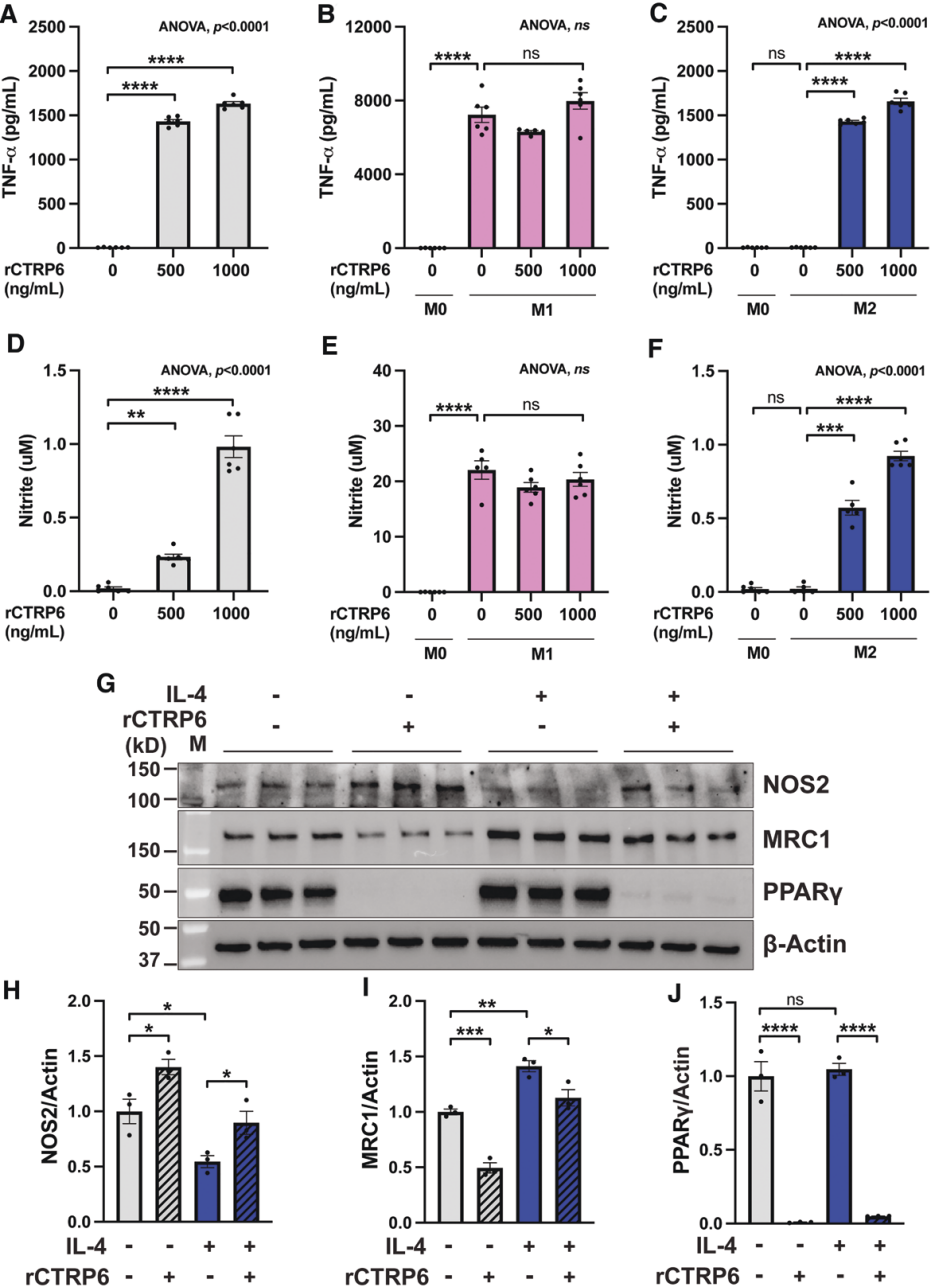
**rCTRP6 protein treatment modulates the expression of marker genes involved in the polarization of BMDMs at the protein level.** (A–C) TNF-α production in BMDMs treated with indicated doses of rCTRP6 protein (*n* = 6/group) was examined by ELISA in M0, M1, and M2 states, respectively. (D–F) NO production in BMDMs treated with indicated doses of rCTRP6 protein (*n* = 6/group) was examined by Griess assay in naïve M0, M1, and M2 states, respectively. Data are shown as the mean ± SEM. Two-tailed Student’s *t*-tests for two-group comparison (M0 vs M1 without rCTRP6; M0 vs M2 without rCTRP6) and one-way ANOVA for multi-group comparison (*P* value indicated in the upper-right corner of the figure) followed by Dunnett’s post hoc test to determine rCTRP6’s effect. ***P < 0.001; ****P < 0.0001. (G) Western blot analysis of NOS2, MRC1, and PPARγ protein in whole cell lysates from naïve M0 state (without IL-4) and polarized M2 state (with IL-4) in conjunction with a 24 hours treatment of 1000 ng/mL rCTRP6. (H–J) Densitometric analysis for Western blot results using ImageJ software (*n* = 3/group). β-actin is used as the internal control. Data are shown as the mean ± SEM. One-way ANOVA followed by Tukey’s post hoc test. *P < 0.05; **P < 0.01; ***P < 0.001; ****P < 0.0001. ANOVA, analysis of variance; BMDMs, bone marrow-derived macrophages; ELISA, enzyme-linked immunosorbent assay; IL-4, interleukin-4; MRC1, mannose receptor C-type 1; NOS2, nitric oxide synthase 2; PPARγ, peroxisome proliferator-activated receptor gamma; SEM, standard error of the mean; TNF-α, tumor necrosis factor-α.

We further validated the changes in marker genes at the protein level. Although *Tnf* mRNA expression did not show dramatic changes, its protein production, measured by ELISA, was significantly enhanced by rCTRP6 treatment, particularly in the M0 and M2 states, with no further increase in the M1 state (Figure 9A–C). Similarly, nitric oxide synthase 2 (NOS2) expression, as indicated by NO production measured by Griess assay, followed a trend similar to TNF-α production (Figure 9D–F). We also confirmed increased NOS2 protein levels in both M0 and M2 states through Western blot analysis (Figure 9G,H). Additionally, M2 marker genes, particularly peroxisome proliferator-activated receptor gamma (PPARγ), were significantly downregulated in both M0 and M2 states (Figure 9G,I,J), underscoring the potent modulatory effect of CTRP6 on macrophage M2 polarization.

### 3.8 Model of CTRP6 modulation of macrophage polarization in adipose tissue

**Figure 10.**
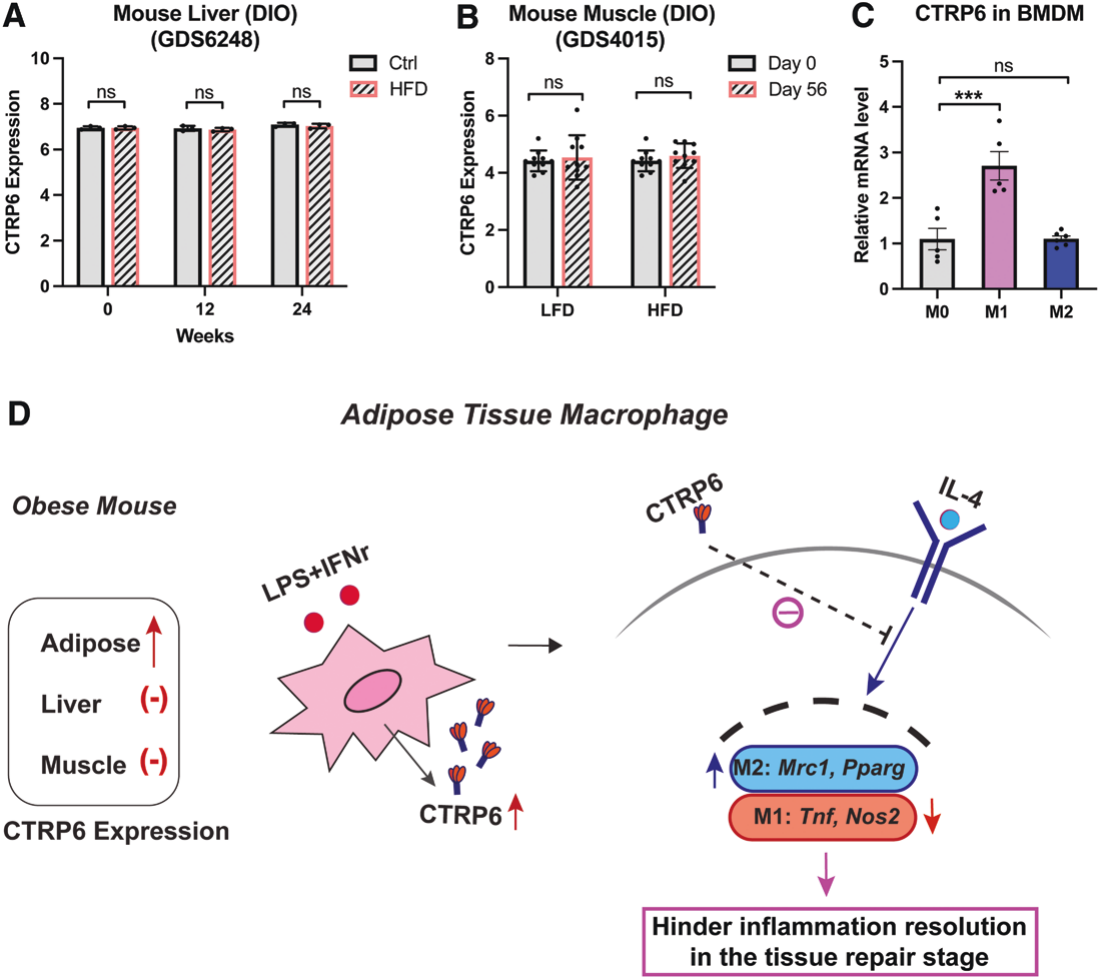
**Model for the role of CTRP6 in obese adipose tissue.** (A) The expression level of CTRP6 in the liver of diet-induced obese (DIO) mouse model at 0, 12, and 24 weeks (*n* = 3/group). The transformed count units were extracted from the GEO database (GDS6248) and analyzed. Data are shown as the mean ± SD. Two-tailed Student’s *t*-tests for two-group comparison. (B) The expression level of CTRP6 in the quadriceps muscle of the DIO mouse model at day 0 and day 56 (*n* = 10/group). The transformed count units were extracted from the GEO database (GDS4015) and analyzed. Data are shown as the mean ± SD. Two-tailed Student’s *t*-tests for two-group comparison. (C) Endogenous CTRP6 mRNA expression levels in BMDMs in the presence of M1 or M2 inducers (*n* = 6/group). Expression levels are normalized to *Ppia*. Data are shown as the mean ± SEM. One-way ANOVA followed by Tukey’s post hoc test. ***P < 0.001. (D) A model for the role of CTRP6 in obese adipose tissue, illustrating how M1 polarization inducers upregulate CTRP6 expression, which subsequently antagonizes IL-4-induced macrophage M2 polarization. This action disrupts the transition to an anti-inflammatory phenotype, exacerbating inflammation by hindering its resolution during the tissue repair stage. ANOVA, analysis of variance; BMDMs, bone marrow-derived macrophages; HFD, high-fat diet; IL-4, interleukin-4; SD, standard deviation; SEM, standard error of the mean.

While the previous study has confirmed increased CTRP6 expression in the adipose tissue of obese mouse models, we explored the potential contribution of other tissues. In obesity, hepatic macrophages, particularly Kupffer cells and recruited monocyte-derived macrophages, become activated and secrete pro-inflammatory cytokines, thereby amplifying liver inflammation ^[42]^. Additionally, several studies have reported that increased macrophage infiltration in skeletal muscle contributes to insulin resistance ^[43,44]^. Thus, we hypothesized that CTRP6 might also be upregulated in the liver or skeletal muscle, potentially contributing to elevated circulating CTRP6 levels in obesity. To test this, we analyzed publicly available microarray data from liver and quadriceps muscle tissues of DIO mouse models. However, we found no significant changes in CTRP6 expression in either tissue (Figure 10A,B), further highlighting adipose tissue as the primary source of increased CTRP6 expression in obesity. Furthermore, we examined endogenous CTRP6 mRNA expression levels in BMDMs. Results show that cells treated with the M1 inducer displayed increased CTRP6 expression, while no change was observed in the M2 state (Figure 10C). Thus, we present a conceptual model illustrating the critical role of CTRP6 in macrophage polarization within the context of obesity in mice (Figure 10D). In the obese state, CTRP6 expression is primarily upregulated in M1 macrophages within adipose tissue. During the early stages of obesity, secreted CTRP6 may contribute to a pro-inflammatory environment by enhancing TNF-α secretion and NO production from adjacent resident macrophages, while concurrently suppressing their M2-associated gene expression, including *Mrc1* and *Pparg*. During the tissue repair phase, CTRP6 appears to antagonize IL-4–mediated M2 polarization by upregulating M1 marker genes (*Tnf*, *Nos2*) and downregulating M2 marker genes (*Mrc1*, *Pparg*). Thus, CTRP6 may exacerbate adipose tissue inflammation by hindering the resolution of inflammation and counteracting the anti-inflammatory effects of IL-4 during tissue repair.

## 4. Discussion

The persistent, unresolved inflammation mediated by macrophages in adipose tissue has been recognized as the underlying factor contributing to pathologies such as insulin resistance and dyslipidemia associated with obesity. However, the mechanisms and specific factors responsible for this dyshomeostasis have been elusive. Although previous studies have shown that CTRP6 is elevated during obesity and functions as a pro-inflammatory factor that further complicates the pathophysiology, the mechanisms of CTRP6 action, however, have not been fully explored. Our present study fills existing gaps by demonstrating that CTRP6 not only promotes the pro-inflammatory M1 phenotype but also significantly impedes the anti-inflammatory M2 phenotype, which aggravates adipose tissue inflammation in obesity.

The previous study attributed the increased CTRP6 expression observed during obese conditions to the stromal vascular fraction of the adipose tissue in mice ^[23]^. Given that macrophages constitute over 50% of the immune cell population in obese adipose tissue ^[10]^, it was logically assumed that macrophages were the main source of the protein. Our results from mining the single-cell/nucleus RNA-seq data published by Emont et al validate this assumption (Figure 1). Moreover, the upregulation of CTRP6 has also been observed in T-cells. Whether and how CTRP6 regulates the function of other immune cells in obese adipose tissue needs to be further explored in future studies.

Studying macrophage polarization in vitro has been challenging due to the complexities associated with the heterogeneity of the macrophage phenotype. In other words, macrophages are highly sensitive to their microenvironments and dynamically exhibit a spectrum of phenotypes between the extremes of pro-inflammatory M1 and anti-inflammatory M2 macrophages ^[12,45]^. To address this issue, we established a benchmark of macrophage marker gene expression profiles for extrinsic induction of polarization using LPS+IFNγ for M1 and IL-4 for M2 phenotypes. The complex nature of these phenotypes is evident in the expression patterns of genes such as *Il1b*, *Il6*, *Il10*, *Ccl2*, and *Arg1*, which are seemingly upregulated during both M1 and M2 induction, with the only differences in the extent (Figure 2). Notably, the strong induction of *Il10* expression during M1 polarization was thought to be responsible for the downregulation of TNF-α, IL-1β, and IL-6 at a later stage, acting as a potent inhibitor of the synthesis of these cytokines ^[46]^. Although *Tgfb* is commonly used as an M2 marker, we could not detect any induction by IL-4 at the mRNA level in our study. Its protein level also did not increase in response to IL-4 ^[47]^, casting doubt on its reliability as an M2 marker gene. However, we did find that the genes *Tnf*, *Nos2*, *Mrc1*, and *Pparg* provide us with a distinctive pattern to identify M1 from M2. The M1 markers *Tnf* and *Nos2* are significantly upregulated during M1 and downregulated during M2, while the M2 markers, *Mrc1* and *Pparg*, are expressed conversely (Figure 2). This overall profile allowed us to evaluate the effect of CTRP6 on macrophage polarization in terms of marker gene expression. It is important to note that the selected marker genes in our study are specifically indicative of mRNA expression levels, conveniently examined through real-time PCR.

In this study, we employed recombinant CTRP6 protein treatment to systematically evaluate its role in macrophage polarization (Figures 3 and 7). The recombinant protein treatment showed a robust effect with increasing doses of protein. To maintain physiological relevance, we employed a concentration closer to the levels observed in obese or diabetic patients (approximately 500 ng/mL) compared with the normal range of 300 to 400 ng/mL ^[32,33,48]^. This approach aimed to reflect conditions more closely aligned with physiological concentrations, in contrast to other studies utilizing higher concentrations such as 10 μg/mL ^[49]^. Moreover, our findings indicate that serum concentration does not significantly contribute to the observed impact of rCTRP6 (Figure 7), thereby validating the consistency of the observed effects.

In RAW264.7 cells, although CTRP6 exhibited slightly different effects under different conditions (M0, M1, and M2), it predominantly promoted M1 and suppressed M2 polarization (Figure 3B). The rCTRP6 protein induced *Tnf* and *Nos2* expression in naïve macrophages (M0) but only led to a 2- to 3-fold increase in mRNA levels. In M1 macrophages, the protein did not significantly affect *Tnf* and had a small impact on *Nos2* expression, suggesting that CTRP6 could slightly intensify the M1 phenotype. Interestingly, CTRP6 had a more pronounced antagonistic effect on IL-4-induced M2 polarization. It seemingly influenced the expression of all our marker genes toward a more inflammatory phenotype with dose-dependent effects on *Tnf*, *Nos2*, and *Mrc1*. Remarkably, the presence of CTRP6 at 1000 ng/mL not only fully counteracted the IL-4-induced 50% inhibition of TNF-α production but also elevated its production to levels even higher than those observed in the M0 state (Figure 4C). Additionally, the simultaneous substantial reduction in MRC1 expression (75% down at the protein level) can have a more significant impact (Figure 4E). Collectively, the dramatic suppression of MRC1 and the upregulation of TNF-α production induced by CTRP6 could aggravate the characteristic low-grade inflammation that persists chronically in obesity. MRC1 protein is a C-type lectin widely expressed by most tissue macrophages and plays a pivotal role in immune homeostasis by scavenging pathogenic glycoproteins through endocytosis and phagocytosis ^[50]^. Given that many pathogenic microbes are coated with mannose-containing structures, strategies optimizing the recycling of this protein in target tissues during infection have become increasingly attractive ^[51]^. On the other hand, the possibility of microbes exploiting CTRP6 to counteract this protein and facilitate their propagation remains unexplored in these infectious diseases.

During the exploration of signaling pathways underlying the impact of CTRP6, we observed that CTRP6 displayed distinct effects on downstream signaling, depending on the macrophage polarization status (Figure 5). In naïve macrophages, CTRP6 effectively downregulated AKT signaling, while it exhibited no significant impact on this signaling in M2 polarization. AKT signaling has been implicated in mediating the suppression of TLR4-induced macrophage activation by adipokines and neuropeptides, such as vasoactive intestinal peptide ^[52,53]^. Given that AKT signaling inherently antagonizes inflammatory responses, a reduction in this signaling indirectly fosters inflammation, contributing to the observed promotion of inflammation in the naïve state. Further studies are needed to investigate whether other inflammatory signals are directly involved in this process. As STAT6 activation is integral to the M2 phenotype ^[54,55]^, the dampening effect of CTRP6 on macrophage polarization toward an anti-inflammatory phenotype is further validated. Previous studies suggest that the activation of ERK1/2 is crucial for an inflammatory M1 response ^[56,57]^. This characteristic is further supported by our findings in macrophages polarized to M2 by IL-4, where ERK1/2 is significantly downregulated. While we also assessed these signaling events in short-term treatments (30 and 60 minutes), no significant changes were observed (data not shown), suggesting that CTRP6 may not directly affect these pathways but rather acts through other mediators.

The polarization signals also induce significant metabolic impacts on macrophages, beyond the molecular changes. For instance, exposure to LPS+IFNγ for the M1 phenotype leads to increased glycolysis and fatty acid synthesis, while the IL-4-mediated M2 response results in elevated OXPHOS and β-oxidation of fatty acids ^[58,59]^. In line with this, our extracellular flux analyses, quantifying the real-time ATP production rates, demonstrated that CTRP6 dose-dependently enhanced glycolytic flux while reducing mitochondrial ATP production, rendering macrophages less energy-efficient (Figure 6A). Although the glycolytic reprogramming is not as pronounced as that induced in M1 macrophages by LPS+IFNγ, the observed trends support the pro-inflammatory tendency of CTRP6 noted in our observations. Furthermore, the diminished mitochondrial activity was validated by staining macrophages for active mitochondria (Figure 6B,C). The consistent reduction in mitochondrial activity induced by CTRP6, irrespective of the macrophage polarization status (naïve or M2), introduces an additional dimension for future detailed investigations into the specific impact of CTRP6 on mitochondrial respiration and energy dynamics. ROS, particularly those derived from mitochondria, are key regulators of macrophage polarization. Previous studies have shown that inhibition of superoxide (O^2−^) production specifically blocks the differentiation of M2 macrophages ^[60]^. Additionally, during tissue repair, macrophages rely on OXPHOS to generate ATP, which supports critical functions in tissue regeneration, including cell migration, proliferation, efferocytosis, and extracellular matrix synthesis ^[61–63]^. Therefore, the CTRP6-mediated suppression of mitochondrial activity may contribute not only to its inhibitory effect on M2 polarization but also to its role in limiting tissue repair.

The monocyte/macrophage-like cell line RAW264.7 has been widely used for over 40 years due to its stable phenotype, expressing macrophage-characteristic genes and surface markers, and consistent functional characteristics, such as phagocytosis and NO production, even across passages (10–30) ^[64]^. However, significant differences have been noted between RAW264.7 and primary BMDMs, particularly in phagosomal composition, as revealed by high-resolution quantitative proteomics ^[65]^. To validate our findings from RAW264.7 cells, we repeated the experiments in primary BMDMs and observed more pronounced effects, notably in the expression of M1 marker *Nos2* and M2 marker *Pparg* at both mRNA and protein levels (Figures 8 and 9). The strong inhibition of PPARγ—a nuclear receptor that dimerizes with retinoic X receptor (RXR) to regulate gene expression—by CTRP6 suggests potential roles in processes such as adipocyte differentiation and glucose and lipid homeostasis, warranting further mechanistic investigation.

The expression of *Ctrp6* gene in mouse adipose tissue has been observed to elevate as early as after 3-day HFD feeding, preceding the upregulation of the inflammatory cytokines such as *Tnf*, *Il1b*, and *Il6* ^[49]^. Further investigation is required to determine whether CTRP6 participates in the early programming of macrophages in animal models. As a strong antagonist of IL-4 and a potent endogenous inhibitor of MRC1, CTRP6 likely affects macrophage functions such as endocytosis and phagocytosis. This impact is not limited to resident M2 macrophages in obese adipose tissue but may extend to newly recruited monocytes and macrophages, collectively hindering the resolution of adipose tissue inflammation. Recently, MRC1-expressing macrophages have been found to be associated with various diseases, including severe chronic obstructive pulmonary disease ^[66]^, inflammatory bowel disease ^[67]^, and many solid cancers ^[68]^. The involvement of CTRP6 in any of these pathophysiology needs to be explored.

## 5. Conclusions

In response to the chronic inflammatory environment in obese adipose tissue, cytokines such as IL-4 are released by tissue-resident immune cells to promote the polarization of macrophages toward an anti-inflammatory M2 phenotype. However, excessive CTRP6 expression, induced by nutritional excess and M1 inducers during obesity progression, acts as a negative regulator that disrupts protective M2 polarization, thereby hindering inflammation resolution. As a result, targeting CTRP6 represents a promising therapeutic approach to tackle the enduring challenges associated with obesity-related pathology.

## Author contributions

J.S.K. and X.L. contributed to the experimental design. J.S.K., E.K., and M.Z.M. performed the experiments. J.S.K. and X.L. analyzed and interpreted the data. D.L. provided technical and conceptual input to the project. J.S.K. and X.L. wrote the paper with inputs from other coauthors. All authors have read and approved the final manuscript.

## Conflicts of interest

All authors declare that they have no conflicts of interest with the contents of this article.

## Funding

This work was supported by the Oklahoma State University Startup Fund granted to X.L. Research reported in this publication was also supported by the National Institute of Diabetes and Digestive and Kidney Diseases of the National Institutes of Health under Award Number R15DK136102. The content is solely the responsibility of the authors and does not necessarily represent the official views of the National Institutes of Health. M.Z.M. is supported by the US-Pakistan Knowledge Corridor PhD Scholarship awarded by the Higher Education Commission of Pakistan.

## Acknowledgments

The authors thank Dr. G. William Wong at the Johns Hopkins University School of Medicine for generously providing the RAW264.7 cells and purified recombinant CTRP6 protein.
